# Fibronectin protein expression in renal cell carcinoma in correlation with clinical stage of tumour

**DOI:** 10.1186/s40364-018-0137-8

**Published:** 2018-07-11

**Authors:** Sandeep Kondisetty, Krishnakumar N. Menon, Ginil Kumar Pooleri

**Affiliations:** 1 Department of Urology, Amrita Institute of Medical Sciences, Amrita Vishwa Vidyapeetham, Ponekkara, Kochi, Kerala India; 2 Center for Nanosciences and Molecular Medicine, Amrita Institute of Medical Sciences, Amrita Vishwa Vidyapeetham, Ponekkara, Kochi, Kerala India

**Keywords:** Fibronectin, Renal cell carcinoma, VHL disease

## Abstract

**Background:**

Carcinogenesis is a multistep process which involves interplay between the tumour cells and the matrix proteins. This occurs by adherence between the tumour cells and proteins in the extracellular matrix. VHL mutation affects through the hypoxia inducible factor (HIF) and causes changes in various tissue proteins like VEGF, PDGF, TGF, Fibronectin and others. As not much literature is available, we aim to quantify the changes of fibronectin protein in renal cell carcinoma (RCC) tissue.

**Methods:**

This Prospective unbalanced case control study was conducted over a period of 18 months from April 2016 to September 2017. The patients undergoing nephrectomy for the diagnosis of RCC were included in the study after obtaining written informed consent. Patients were excluded from study, if normal renal tissue could not be identified in the resected kidney and if the artery clamp time to retrieval of tissue was more than 30 min. Fibronectin protein is estimated in the tumour tissue by gel electrophoresis and western blotting which is compared with that of normal kidney tissue of the same kidney. Results have been expressed as absolute values with standard deviation and relative expression (RE).

**Results:**

Of the 21 patients analysed 15 showed an increase in fibronectin expression in the renal tumour tissue while 6 did not. The mean expression of Fibronectin protein has increased 1.5 times in the tumour tissue when compared with the normal tissue. The increase was 1.54 times in early tumours compared to 1.37 times in advanced tumours of RCC.

**Conclusions:**

Fibronectin showed a 1.5 times increase in the tumour compared to normal. This increase is more in Stage 1&2 tumours when compared to the Stage 3&4 tumours.

**Electronic supplementary material:**

The online version of this article (10.1186/s40364-018-0137-8) contains supplementary material, which is available to authorized users.

## Background

Renal cell carcinoma (RCC) is the most common renal malignancy and accounts to 3% of all human cancers [1]. There is a substantial increase in the RCC in the recent few years. The incidence of RCC is more in Western countries compared to India and other Eastern countries. There were about 12,500 new cases of renal tumours in the UK in 2014 with an incidence of 25/100,000. In India, the incidence of RCC was estimated at 2/100,000 population among males and about 1/100,000 population among females [2].

With increasing incidence, to combat the disease new modalities of treatment are mainly targeting the VEGF and VHL pathways. Von Hippel-Lindau (VHL) disease is an autosomal dominant hereditary disorder characterized by many tumours including clear cell renal cell carcinoma [3]. The frequency of VHL mutation event ranges from 46 to 82% of sporadic cases of RCC. Vikkath et al. has screened 30 VHL patients and found that RCC is the presenting manifestation in 7 (22.6%) patients [4]. VHL mutation affects through the hypoxia inducible factor (HIF) and causes changes in various tissue proteins like VEGF, PDGF, TGF, Fibronectin and others. As not much is known about the changes in FN in renal cancers, we had decided to study FN protein in early and advanced stages of RCC. Already drugs targeting VEGF are available but there is no drug in clinical use targeting FN. Other drugs in clinical use are tyrosine kinase inhibitors and mTOR inhibitors.

Carcinogenesis is a multistep process which involves interplay between the tumour cells and the matrix proteins. This occurs by adherence between the tumour cells and proteins in the extracellular matrix. Among the many extracellular matrix proteins which were identified, Fibronectin (FN) a glycoprotein seems to be involved in adhesion and migration processes of the cancer cells [5]. He et al. [6] was investigating the changes which happen by VHL mutation in RCC tumour tissue. He found in a RCC patient there was HIF-1α diffuse granular staining in cytoplasm of tumour cells if there is presence of von Hippel-Lindau (VHL) gene mutation and no staining in the absence of VHL mutation. At the same time distribution of FN in tissue changes in the presence of VHL mutation. In presence of VHL mutation the matrix fibronectin has decreased with presence of FN at varying levels in the cytoplasm of tumour cells. In the absence of VHL mutation in the RCC the matrix FN is strong with absence of FN in the tumour cell cytoplasm [6]. This suggests that FN protein changes in the tumour cells and the surrounding matrix play a role in all patients of RCC especially with VHL mutations.

## Methods

### Patients

This Prospective unbalanced case control study was conducted in the Department of Urology at Amrita Institute of Medical Sciences, Kochi, India over a period of 18 months from April 2016 to September 2017. The patients undergoing Partial / Radical Nephrectomy for the diagnosis of RCC were included in the study after obtaining written informed consent. Patients were excluded from study, if normal renal tissue could not be identified in the resected kidney particularly in partial nephrectomy and if the artery clamp time to retrieval of tissue was more than 30 min. Tumor tissue samples were grouped based on TNM (tumour-node-metastasis) classification 7th edition as proposed by Union International Cancer Control (UICC) and American Joint Committee on Cancer (AJCC) in 2009 and nuclear grading according to the Fuhrman grading system [7]. Histological types were based on the consensus classification of renal cell neoplasia [8]. As the tumour stromal percentage (TSP) is coming up as novel independent predictor in many solid organ tumours we have attempted to classify the tumours based on stromal content into 3 groups. The first is less than 10%, second 10–50% and lastly more than 50%. Gujam et al. has studied on the outcome of breast cancer based on different tumour stromal percentage [9]. Patient data was collected from the hospital electronic medical records system and subsequently analysed using a microsoft excel program. The study was approved by the institutional ethics committee and scientific review committee.

### Tissue specimens

The renal tissue was collected immediately after surgery for RCC by taking a small fragment minimum of 5 mm from macroscopically appearing tumour tissue and another from the surrounding normal appearing tissue which is the control for protein estimation analysis for further fibronectin protein level determination using immunoblotting experiments. After confirming the tissue by frozen section with haematoxylin and eosin staining, the tissue was labelled and immediately stored at − 80 degrees Celsius within 30 min from devascularisation. The tissue was homogenised with gentle MACS Dissociator with the lysate buffer. Lysate buffer was made with 6 g of Urea, 2.5 g of SDS in 50 ml of PBS. To 200 mg of the tissue 1.7 ml of Lysate Buffer was added and homogenized with 40 μl of Protease Inhibitor 50X. Then the total protein was estimated in both the tissues by using the Bradford protein estimation method [10]. The mean protein in the tumour was 8.95 ± 1.36 mg/ml while that in the normal renal tissue was 8.29 ± 2.39 mg/ml. This shows that there is no significant difference in the total protein of RCC tumour or the renal normal tissue (*p* value = 0.23).

### Laboratory procedure

The homogenate was used for further fibronectin analysis. Gel electrophoresis (SDS-PAGE) was done using 4–15% Mini-PROTEAN® TGX™ Precast Protein Gels, 15-well, 15 μl and Mini-PROTEAN® Tetra Vertical Electrophoresis Cell. 30μgm of protein was loaded in each well for the electrophoresis. The gels were western blotted for further probing using specific antibodies against fibronectin. The antibody used was rabbit polyclonal IgG antibody from Santa Cruz Biotechnology Fibronectin Antibody (H-300). This antibody recognises the protein FN at 220 kDa. Following chemiluminiscent reaction the membrane was exposed and image was captured using ChemiDoc XRS+ system. The expression of proteins was analysed quantitatively by densitometric analysis using ImageJ software (National Institutes of Health) which is one of free software available. Actin staining (Additional file 1) was done to normalize the immunoblots and served as loading controls. Results have been expressed as absolute values with standard deviation and relative expression (RE) considering the ratio between the optical densities of protein band under study to corresponding actin band optical density multiplied by the average of actin bands.

### Statistical analysis

All data collected was entered in Microsoft excel program and analyzed by using IBM SPSS software version 20. All the continuous variables were expressed using mean and standard deviation and categorical variables were presented using frequency and percentage. To test the statistical significance of mean protein changes from normal to tumour based on tumour size, clinical stage, furhman’s grade, tumour stromal percentage and histological type, Wilcoxon signed ranks test was used. To test the statistical significant changes in tumour between Stage 1&2 and Stage 3&4 Mann-Whitney U test was used. To test the statistical significant difference in increase or decrease protein between tumour and normal Chi-square test/ Fisher’s exact test was used. A *p* value < 0.05 was considered statistically significant.

## Results

A total of 21 patients were included in the study, the Fibronectin levels were quantified from both the tumour tissue and the adjacent normal kidney tissue. The clinicopathological data of all the patients included in the study were given in Table 1.

**Table 1:** Clinicopathological data regarding the patients from whom the sample both tumour and normal was collected

	Clinicopathological variables	
	Number of patients (*n* = 21)	Percentage of patientspercentage of patientspercentage of patients
Age (*n* = 21)		
< 50	6	28.57
50–60	8	38.1
60–70	4	19.05
> 70	3	14.28
Sex (*n* = 21)		
Male	14	66.67
Female	7	33.33
Surgery (*n* = 21)		
Partial nephrectomy	10	47.61
Radical nephrectomy	11	52.39
Histology type by H & E staining (*n* = 21)		
Clear cell carcinoma	15	71.43
Papillary carcinoma	3	14.29
Chromophobe carcinoma	2	9.52
Others / Multilocular cystic renal cell neoplasm	1	4.76
Furhmann’s grade by H & E staining (*n* = 19)		
1	1	5.26
2	12	63.16
3	6	31.58
T – Primary tumour (*n* = 21)		
T1a	5	23.81
T1b	9	42.86
T2b	2	9.52
T3a	5	23.81
Lymph node metastasis (*n* = 21)		
Absent	21	100
Distant metastasis (*n* = 21)		
Absent	20	95.24
Present	1	4.76
Stage (*n* = 21)		
I	14	66.67
II	2	9.52
III	4	19.05
IV	1	4.76
Tumour stroma percentage / TSP (*n* = 21)		
< 10%	20	95.24
> 50%	1	4.76

**Fig. 1:**
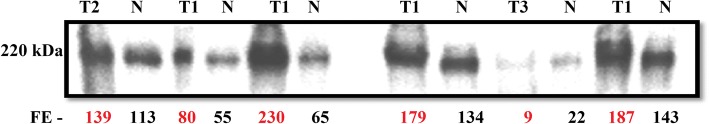
Western Blot showing-Fibronectin Protein Bands resolved at 220 kDa in SDS PAGE. Fibronectin Expression(FE) of tumour showed in red and normal tissue showed in black. T1- Tumour stage I, T2- Tumour stage II, T3- Tumour stage III

**Fig. 2:**
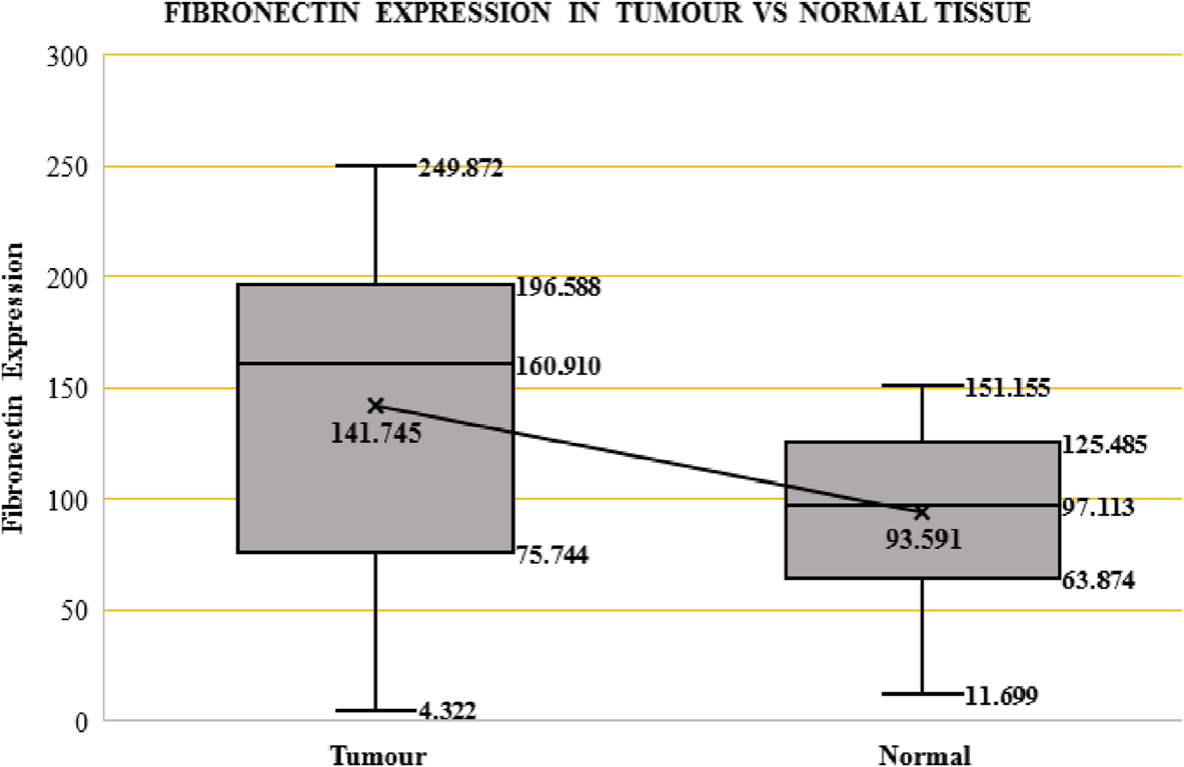
Box-Whisker Plot showing changes in Fibronectin Expression in Tumour vs Normal Tissue

Western blot analysis to detect Fibronectin (FN) levels (Fig. 1) showed that mean expression levels of fibronectin in tumour tissue is 141.75 ± 75.11 while in normal tissue the mean expression is 93.59 ± 38.29 which is statistically significant (*p* value = 0. 002) (*n* = 21) (Fig. 2). The mean expression of Fibronectin protein has increased 1.5 times in the tumour tissue when compared with the normal tissue. Of the 21 patients analysed 15 showed an increase in fibronectin expression in the renal tumour tissue while 6 did not. The increase is noted in both tumours with clear cell histology and also the non-clear cell tumours.

The mean expression of fibronectin in Clear cell tumours is 131.62 ± 80.86 while in normal tissue the mean expression is 85.14 ± 38.13 which is statistically significant (*p* value = 0.023). The mean expression of fibronectin in non-clear cell tumours is 167.06 ± 56.41 while in normal tissue the mean expression is 114.72 ± 32.29 which is also statistically significant (*p* value = 0.046) (Table 2). The increase is 1.55 times in clear cell tumours and 1.46 times in non-clear cell tumours. The mean expression of fibronectin in tumours with tumour stromal percentage (TSP) < 10% is 141.9 ± 77.06 when compared to normal tissue which has 92.62 ± 39.02. The increase in these tumours is 1.53 times which was statistically significant (*p* value = 0.002). The mean expression of fibronectin in tumour (*n* = 1) with tumour stromal percentage (TSP) > 50% is 138.66 when compared to normal issue which has 113. The increase in this tumour is 1.23 times only but cannot draw any conclusions as this was only one in the group.

**Table 2:** Changes in Fibronectin protein in RCC tumour against normal renal tissue

	Fibronectin expression			
	RCC tissue	Normal renal tissue	Relative increase	*p*-value
All specimens n = 21	141.75 ± 75.11	93.59 ± 38.29	1.5	0.002
Clear cell tumours *n* = 15	131.62 ± 80.86	85.14 ± 38.13	1.55	0.023
Non clear cell tumours *n* = 6	167.06 ± 56.41	114 ± 32.29	1.46	0.046
Stage I and II *n* = 16	159.18 ± 65.47	103.21 ± 325	1.54	0.004
Stage III and IV *n* = 5	85.95 ± 83.79	62.83 ± 42.62	1.37	0.686

The mean expression of fibronectin in Stage 1 &2 tumours is 159.18 ± 65.47 while in normal tissue the mean expression is 103.21 ± 32.5 which is statistically significant (*p* value = 0. 004). The mean expression of fibronectin in Stage 3 & 4 tumours is 85.95 ± 83.79 while in normal tissue the mean expression is 62.83 ± 42.61 which is not statistically significant (*p* value = 0. 686) (Table 2). The increase was 1.54 times in early tumours compared to 1.37 times in advanced tumours of RCC. In early renal tumours fibronectin is increased in 13 cases but decreased in 3 cases while in advanced renal tumours it is increased in only 2 cases but decreased in 3 cases (*p* = 0.115, Fisher’s exact test).

## Discussion

FN plays a critical role in mediating cell adhesion and migration, and also has a role in the process of apoptosis in certain tissues [11]. Waalkes et al. [12] compared 109 RCC tissue with 86 adjacent normal tissue by doing mRNA detection and concluded a notable increase in expression of fibronectin mRNA in RCC when compared to normal renal tissue. In their study both organ-confined and advanced disease showed significant higher levels of fibronectin mRNA expression compared to normal renal tissue. At the same time they concluded that the increase in FN is higher as the stage of disease advances. In our study the protein expression was found to be increased in all stages of renal tumours but the early stages showed a higher expression of fibronectin when compared to advanced tumours which is a new finding. This is in contrast to what was shown before by Waalkes et al. [12]. It has to be noted that presence of mRNA not necessarily always leads to translation into protein. Therefore identification of FN protein presence is more relevant in the functional context.

Steffens et al. [13] collected 270 clear cell RCC tissue specimens of patients who had surgery for the disease and did immunohistochemistry on the tissue. They found to have no significant association between cytoplasmic FN staining and patient characteristics such as age, gender, tumor differentiation and visceral metastasis. As they evaluated survival rates both tumor-specific as well as overall survival rate in 153 patients, they concluded mortality rate in patients with increased cytoplasmic FN expression, indicating a possible role of FN in the prognosis of RCC. This is a large volume study after the He et al. [6] who also used immunohistochemistry of the tumour tissue to determine the FN content. Indeed, in our study we show that early tumors tend to have higher expression of FN compared to late stage indicating that FN may play a role in the progression of tumor and that once it reaches a late stage levels do not change.

Till date there are no drugs against Fibronectin in clinical use. Pharmacological agents targeting FN will be a hope of future as animal experiments by Chaves et al. with endostatin gene therapy shows a promising result [14]. This group has studied the murine model of metastatic RCC in lungs. After endostatin gene therapy the FN levels were 80% less than the control groups who did not receive the therapy. In this model they used densitometric quantification of the immunoblot for the estimation of FN and a similar quantification technique was used by us. In this context, identification of differential expression of FN in normal vs early stage tumor in our study may have potential prognostic value in preferential endostatin therapy to patients who are overexpressing FN.

## Conclusion

On western blot protein analysis of 21 RCC tissue and the normal kidney tissue of the same patient, fibronectin showed a 1.5 times increase in the tumour compared to normal. This increase is more in Stage 1&2 tumours when compared to the Stage 3&4 tumours. Although the number is the limitation the relative decrease in FN in advanced disease may be interesting finding for further refinement.

## Additional file

Additional file 1: Actin bands seen at 45 kDa which were used as loading controls for calculating the expression of FN in tumour and normal tissues. (JPG 426 kb)

## Abbreviations

FN: Fibronectin
RCC: Renal cell carcinoma
SDS-PAGE: Sodium dodecyl sulfate polyacrylamide gel electrophoresis
TSP: Tumour stromal percentage
VEGF: Vascular endothelial growth factor
VHL: Von Hippel-Lindau
