# Supplementary figures and images for: Fibronectin protein expression in renal cell carcinoma in correlation with clinical stage of tumour
Source: Biomark Res. 2018 Jul 11;6:23. doi: 10.1186/s40364-018-0137-8 (PMC6042246; doi:10.1186/s40364-018-0137-8)

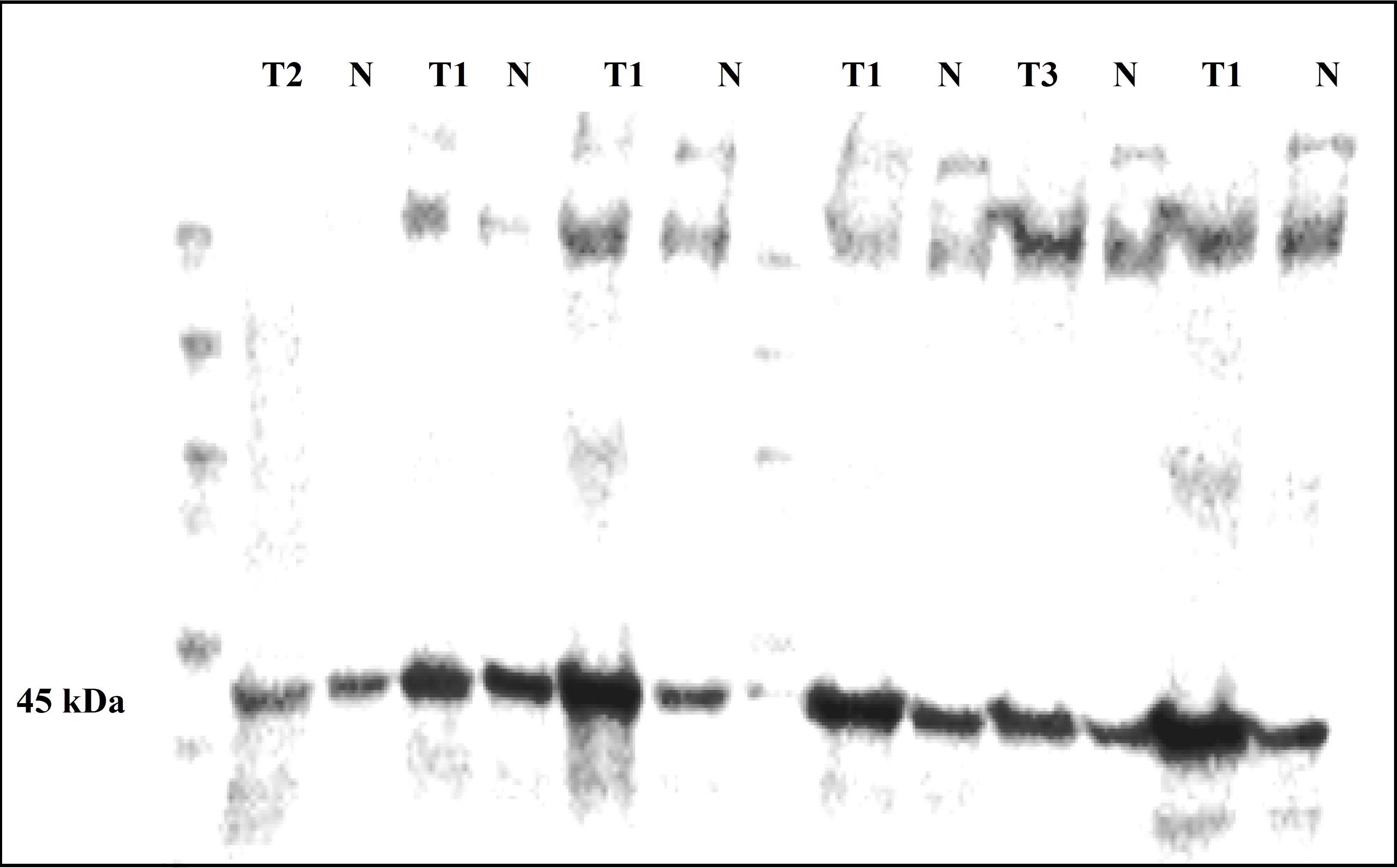

Supplement: Supplementary file 1 — Actin bands seen at 45 kDa which were used as loading controls for calculating the expression of FN in tumour and normal tissues. (JPG 426 kb) [file 40364_2018_137_MOESM1_ESM.jpg]
